# Pure akinesia with gait freezing: a clinicopathologic study

**DOI:** 10.1186/s40734-017-0063-1

**Published:** 2017-10-17

**Authors:** Ahmad Elkouzi, Esther N. Bit-Ivan, Rodger J. Elble

**Affiliations:** 1 Department of Neurology, Southern Illinois University School of Medicine, PO Box 19645, Springfield, IL 62794-9645 USA; 2 Department of Pathology, Southern Illinois University School of Medicine and Memorial Medical Center, Springfield, IL USA

**Keywords:** Akinesia, Bradykinesia, Hypokinesia, Progressive supranuclear palsy, Freezing of gait

## Abstract

**Background:**

Pure akinesia with gait freezing is a rare syndrome with few autopsied cases. Severe freezing of gait occurs in the absence of bradykinesia and rigidity. Most autopsies have revealed progressive supranuclear palsy. We report the clinical and postmortem findings of two patients with pure akinesia with gait freezing, provide video recordings of these patients, and review the literature describing similar cases. We also discuss bradykinesia, hypokinesia and akinesia in the context of this clinical syndrome.

**Case presentation:**

Two patients with the syndrome of pure akinesia with gait freezing were examined by the same movement disorder specialist at least annually for 9 and 18 years. Both patients initially exhibited freezing, tachyphemia, micrographia and festination without bradykinesia and rigidity. Both autopsies revealed characteristic tau pathology of progressive supranuclear palsy, with nearly total neuronal loss and gliosis in the subthalamus and severe neuronal loss and gliosis in the globus pallidus and substantia nigra. Previously published postmortem studies revealed that most patients with this syndrome had progressive supranuclear palsy or pallidonigroluysian atrophy.

**Conclusions:**

Pallidonigroluysian degeneration produces freezing and festination in the absence of generalized slowing (bradykinesia). Freezing and festination are commonly regarded as features of akinesia. Akinesia literally means absence of movement, and akinesia is commonly viewed as an extreme of bradykinesia. The pure akinesia with gait freezing phenotype illustrates that bradykinesia and akinesia should be viewed as separate phenomena.

**Electronic supplementary material:**

The online version of this article (doi:10.1186/s40734-017-0063-1) contains supplementary material, which is available to authorized users.

## Background

Steele, Richardson and Olszewski described the classic phenotype of progressive supranuclear palsy (PSP): vertical gaze palsy, postural instability, neck rigidity, dysarthria, pseudobulbar palsy and frontal dysexecutive syndrome, but they acknowledged that further observations may broaden the clinical spectrum of PSP [1]. Several clinical phenotypes of PSP have since been described, and overlapping phenotypes are common [2, 3]. One rare phenotype is pure akinesia with gait freezing (PAGF) [3, 4]. Although this phenotype is not specific for PSP, there is growing postmortem evidence that most cases have PSP pathology. We now report the autopsy findings of two patients with PAGF, and we present videos of these patients to illustrate the clinical features and progression of this disorder. To our knowledge, these are the first published videos of PAGF in patients with autopsy-confirmed PSP. We also briefly discuss the terminology of bradykinesia, hypokinesia and akinesia in the context of PSP-PAGF.

## Case presentations

Two patients with PAGF were examined at least annually by the same movement disorder specialist (RJE) from the time they were first seen in his clinic. Both patients were videotaped one or more times with their informed written consent, approved by our human subjects committee. Both patients underwent autopsy (brain only). Gross examination of the brains was conducted, and sections from grossly normal and abnormal regions were taken for histologic examination. After study of routine hematoxylin and eosin stained slides, the cases were scrutinized for PSP and other neurodegenerative diseases using immunohistochemical stains for abnormal tau, alpha-synuclein, synaptophysin and amyloid aggregates in Patient 1 and for abnormal tau and amyloid in Patient 2. The two autopsies were performed by different neuropathologists more than 10 years apart, and no attempt was made to compare the two cases quantitatively or to compare these cases with other cases of PSP.

On February 1, 2017, we searched PubMed for cases of pure akinesia with gait freezing in which the diagnosis was established by autopsy, biopsy or genetic testing. The search criteria *((((((pure akinesia) OR freezing gait) OR gait ignition failure) OR primary akinesia) OR gait freezing)) AND pathology* produced the abstracts of 186 papers. Forty-nine relevant articles were reviewed in detail, resulting in 14 reports with adequate clinical and pathologic/genetic descriptions. All but one [5] were in English.

Our patients initially presented with the syndrome of PAGF: start hesitation and freezing of gait, festination of repetitive limb movement, festination of speech (tachyphemia), “fast micrographia” [6], and no rescue response on the pull test. Neither patient responded to levodopa. Both learned to break freezing with deliberate motor tricks such as stepping over the handle of a cane or a laser beam projected onto the floor (*kinesia paradoxica* [7]). Rigidity, bradykinesia, supranuclear gaze palsy, impaired balance, and other features of classic PSP appeared after more than 5 years of “pure akinesia”. Neither patient exhibited dementia. The clinical course and postmortem findings are now summarized for each patient.

### Patient 1

At age 66, this man first noticed freezing when dancing and when getting in or out of his car. Within a year, he noticed freezing when initiating gait and when walking through thresholds, but he otherwise walked normally. He also noticed micrographia and festinating speech (tachyphemia). There was no tremor, cognitive impairment, dysphagia, or problems with urinary bladder or bowel function. He had no other medical problems except mild hypertension, controlled with a thiazide diuretic, and he had no family history of neurological problems. He tried carbidopa-levodopa 50/200 four times daily without benefit.

He came to our clinic at age 69. His Mini Mental State Exam was 30, and his eye movements were normal clinically and by quantitative oculography. There was festination and decrement in volume/amplitude of his speech and repetitive hand movements, but he had no bradykinesia (slowness of movement), rigidity, tremor or ataxia. His handwriting was barely legible due to micrographia. There was occasional freezing on initiation of gait and frequent freezing when encountering doorways and other thresholds, but he otherwise walked normally (Additional file 1: Video 1). He had no rescue response on the pull test, but he had no difficulty tandem walking. His brain MRI revealed a few small foci of T2 signal scattered in the cerebral white matter, an empty sella, but no other abnormalities (no brainstem atrophy).



**Additional file 1: Video 1.** This is patient 1 at age 69, three years after the onset of symptoms. He had severe freezing of gait, but his gait was otherwise normal. Tandem walking was normal. He had no rescue response to the pull test. He exhibited festination and decrement in volume/amplitude of his speech and repetitive hand movements, but he had no bradykinesia, rigidity, or tremor. His eye movements were normal.


At age 76, he exhibited upward gaze paralysis, slow downward saccades, eyelid paratonia, moderate generalized bradykinesia and rigidity, severe tachyphemia, and increased freezing (Additional file 2: Video 2). There was no response to amantadine, galantamine, selegiline, ropinirole, duloxetine, pergolide or another trial of carbidopa-levodopa. At age 79, he could not stand or walk without assistance. He had eye opening apraxia and blepharospasm. By age 84, he had complete vertical gaze paralysis, severe generalized rigidity and bradykinesia, and inability to stand and walk. A third trial of carbidopa-levodopa produced no benefit. He lived at home with his wife until his death at age 87.

**Fig. 1:**
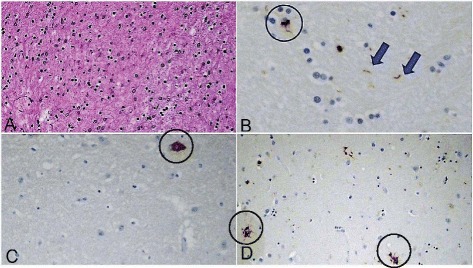
**a**: hematoxylin-eosin stain of the subthalamic nucleus showing severe neuronal loss and gliosis. **b**: tau stain of cerebellar white matter revealing coiled bodies (circle) and neuropil threads (arrows). Granular neuronal inclusions in the globus pallidus (**c**) and scattered tufted astrocytes in the putamen (**d**) revealed by tau stain

**Fig. 2:**
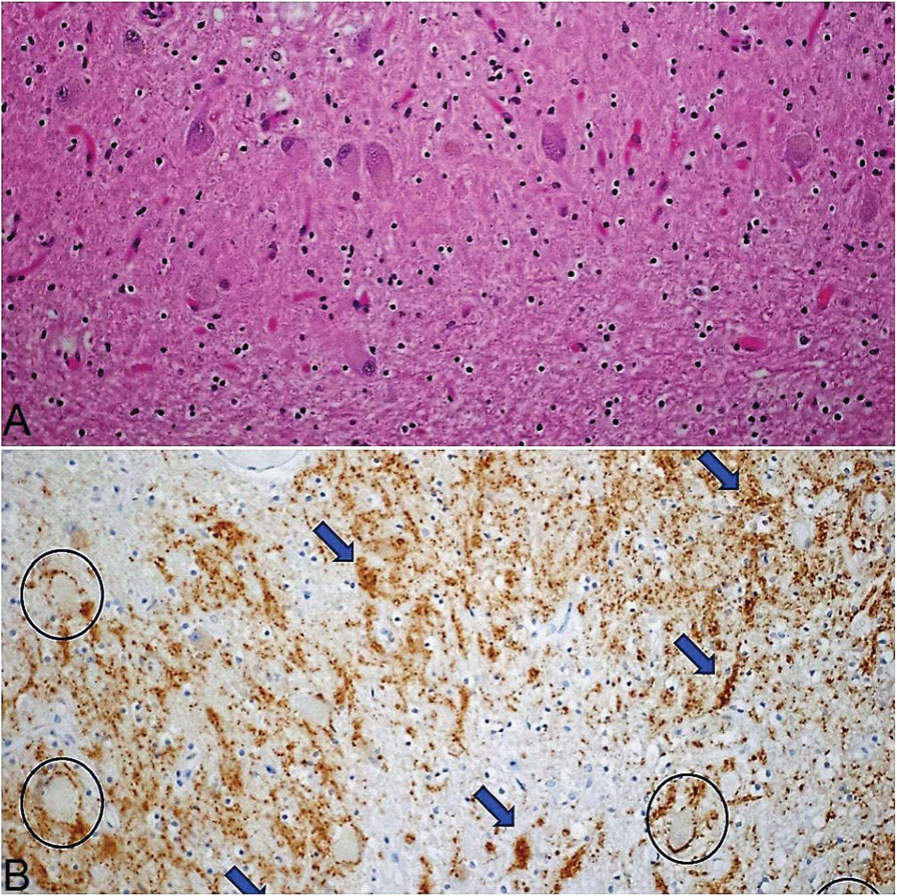
**a**: hematoxylin-eosin stain of the dentate nucleus showing moderate neuronal loss and gliosis. **b**: synaptophysin stain of the dentate nucleus showing grumose degeneration (arrows) and normal synaptic staining around neurons (circles)



Gross examination of the brain revealed severe atrophy of the subthalamic nucleus, moderate atrophy of the globus pallidus, and pallor of the substantia nigra. Fixed brain weight was 1430 g. Histologic sections demonstrated nearly complete neuronal loss in the subthalamic nucleus, severe neuronal loss in the globus pallidus and substantia nigra, moderate neuronal loss in the superior colliculi, and mild neuronal loss in the locus ceruleus and dentate nucleus. Immunohistochemical stains for tau revealed scattered tufted astrocytes, rare coiled oligodendroglial inclusions, and globose neurofibrillary tangles (Figs. 1 and 2). Synaptophysin immunohistochemical staining revealed mild grumose degeneration of the dentate nucleus. Alpha synuclein and Congo red stains were negative.

**Additional file 2: Video 2.** Six years after video 1, patient 1 exhibited generalized bradykinesia and rigidity, increased freezing, upward gaze paresis, slow downward saccades, eyelid paratonia, and severe tachyphemia.


### Patient 2

This woman first noticed micrographia, tachyphemia and falls due to freezing at age 65. There was no bradykinesia (slow movement), rigidity or tremor. An MRI revealed a left parietal meningioma but no other abnormalities (no brainstem atrophy). Resection of the meningioma at age 66 produced no clinical change. By age 71, she was falling at least weekly. She had mild bradykinesia, but no rigidity or tremor (Additional file 3: Video 3). There was festination and decrement in speech and repetitive hand movements, severe micrographia, and normal eye movements. Her Mini Mental State score was 30. She had no rescue response on the pull test, but she could tandem walk six steps without sidestepping. She did not respond to carbidopa-levodopa, rasagiline, memantine, pramipexole, galantamine, or amantadine. There was no family history of similar problems.

She died of lung cancer at age 74. During the last year of her life, she began exhibiting generalized motor slowing, mild slowing of upward and downward saccades, and frequent ocular square wave jerks during fixation. Gross postmortem examination revealed marked atrophy of the subthalamic nucleus and globus pallidus bilaterally and pallor of the substantia nigra. Fixed brain weight was 1150 g. Tau immunohistochemistry demonstrated globose tangles in the substantia nigra, globus pallidus, subthalamic nuclei, inferior olives, dentate nucleus, and pontine reticular formation. Tufted astrocytes were present in the striatum. The right hippocampal gyrus contained a microscopic focus of poorly differentiated carcinoma. Congo red stain was negative.



**Additional file 3: Video 3.** Patient 2 at age 71 had a six-year history of tachyphemia, micrographia and freezing gait. Her examination revealed tachyphemia, festination and decrement in repetitive hand movements, cautious gait, freezing, absent postural rescue response, and normal tandem gait.


## Discussion

Freezing of gait is defined as “brief, episodic absence or marked reduction of forward progression of the feet despite the intention to walk” [8] and is associated with festination of gait, repetitive finger movements and speech (tachyphemia) [9]. Freezing of gait has been described in PSP, Parkinson disease, multiple system atrophy, normal pressure hydrocephalus, and other diseases affecting the frontal lobes or basal ganglia bilaterally [10]. Patients with freezing in the absence of dementia, bradykinesia, rigidity and impaired balance are rare [11].

Patients presenting with freezing of gait have been variously labeled as having isolated freezing of gait, gait ignition failure, and primary progressive freezing of gait [12]. Williams and coworkers [4] coined the term pure akinesia with gait freezing (PAGF) to describe a rare phenotype of PSP [4, 13, 14] that was initially described by Imai and Narabayashi [15]. This phenotype consists of the insidious onset of gait freezing, tachyphemia, and micrographia, and there is no response to levodopa and no history or neuroimaging evidence of subcortical vascular disease (Binswanger disease). For at least 5 years, there is no rigidity, tremor, dementia or supranuclear ophthalmoplegia. Our patients fulfilled all of these criteria. Both patients ultimately developed additional clinical signs of PSP, and autopsy confirmed the diagnosis of PSP. Both patients illustrate the relatively slow clinical course of PSP-PAGF to immobility, compared to classic PSP [4].

Most patients with the clinical syndrome of PAGF have pathology that is characteristic of PSP [4, 16–18], and they exhibit a pattern of striatal dopaminergic loss that is characteristic of PSP [13]. Less commonly, PAGF is caused by Lewy body disease [19], pantothenate kinase 2 gene mutation [20], pallidonigroluysian atrophy (PNLA) [21–23], and primary lymphoma primarily affecting the basal ganglia [24]. PNLA is the second most common cause of PAGF, and its relationship to PSP is still debated (Table 1) [23, 25, 26].

**Table 1:** Published cases of PAGF for which diagnosis was established by autopsy, biopsy, or genetic testing

Report	Number and gender of patients	Age of onset (years)	Diagnosis
Compta et al. [18]	1 woman	67	Progressive supranuclear palsy
Williams et al. [4]	5 men 2 women	44–78 (mean 61)	6 Progressive supranuclear palsy 1 Lewy body Parkinson disease
Matsuo et al. [17]	2 men	62, 72	Progressive supranuclear palsy
Mizusawa et al. [30]	1 woman 3 men	55 62, 65, 70	Progressive supranuclear palsy
Facheris et al. [27]	1 man	57	Progressive supranuclear palsy
Yoshikawa et al. [29]	1 woman	58	Progressive supranuclear palsy
Homma et al. [5]	1 woman	54	Progressive supranuclear palsy
Ahmed et al. [23]	7 men 1 woman	57.1 ± 3.1	Pallidonigroluysian atrophy
Konishi et al. [21]	1 man	60	Pallidonigroluysian atrophy
Factor et al. [22]	1 man 1 woman	74 77	Pallidonigroluysian atrophy Lewy body disease
Katayama et al. [38]	1 man	31	Pallidonigral atrophy
Quinn et al. [19]	1 man	53	Lewy body Parkinson disease
Molinuevo et al. [20]	1 man and 1 woman (siblings)	35 and 51	Pantothenate kinase 2 gene mutation
Pramstaller et al. [24]	1 man	75	Primary CNS lymphoma affecting the basal ganglia bilaterally

**Fig. 3:**
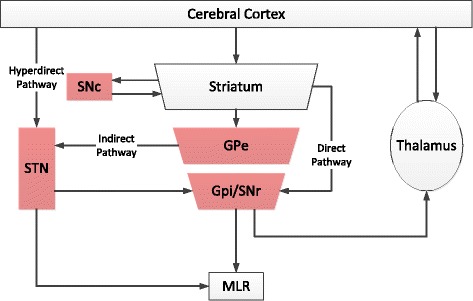
Schematic diagram of the cortical-basal ganglia-thalamocortical loop. The areas of greatest neurodegeneration in PAGF caused by PSP and PNLA are shown in red. The hyperdirect pathway (cortex-STN-GPi), direct pathway (cortex-striatum-GPi) and indirect pathways (cortex-striatum-GPe-STN-GPi) are all interrupted by pathology, thereby precluding modulation of the thalamus and midbrain locomotor region (MLR), which includes the pedunculopontine and cuneiform nuclei. GPe: globus pallidus externa, GPi: globus pallidus interna, SNc: substantia nigra pars compacta, SNr: substantia nigra pars reticulata, STN: subthalamic nucleus

The pathology in our patients was similar to other reported cases of PSP-PAGF [18, 27, 28]. Greatest pathology occurred in the subthalamic nucleus, globus pallidus, and substantia nigra. This anatomical distribution of neurodegeneration has been described consistently in previously reported cases of PAGF caused by PSP and by PNLA [17, 23, 28–30]. Therefore, the direct, indirect and hyperdirect pathways of the basal ganglia [31] are affected in the initial stages of PSP-PAGF and PNLA-PAGF, and this produces severe freezing with little or no bradykinesia (i.e., slowness) (Fig. 3).

The absence of bradykinesia (slow movement) in PSP-PAGF, despite severe freezing and festination, is quite impressive when one considers the fact that patients with PSP-PAGF have severe nigrostriatal dopaminergic loss [13]. The absence of bradykinesia, rigidity and tremor in PSP-PAGF has been attributed to a pallidotomy effect of disease-related destruction of the pallidum [15], but a better understanding of the pathophysiology of bradykinesia is needed to fully explain the PSP-PAGF phenotype.

At issue in this discussion is the definition of bradykinesia, which literally means slow movement and is widely regarded as a classic sign of Parkinson disease. The London Brain Bank definition of bradykinesia conflates slowness with decrement of movement amplitude: “slowness of initiation of voluntary movement with progressive reduction in speed and amplitude of repetitive actions” [32]. This definition of bradykinesia, in the context of Parkinson disease, was retained in the International Parkinson and Movement Disorder Society (MDS) diagnostic criteria for Parkinson disease: “Bradykinesia is defined as slowness of movement AND decrement in amplitude or speed (or progressive hesitations/halts) as movements are continued” [33]. The authors of the MDS criteria acknowledged that “Bradykinesia as defined here combines with one term the definitions of bradykinesia (slowness) and akinesia/hypokinesia (decreased movement amplitude); both are generally present on examination, although not always simultaneously (i.e., patients cannot move at normal speed with normal amplitude)”. In discussions of bradykinesia, it is frequently unclear whether a clinician is using the pure definition of bradykinesia (i.e., slowness) or the London Brain Bank/MDS definition of bradykinesia for Parkinson disease (slowness and decrement) [34]. The PAGF phenotype illustrates that decreased movement amplitude can occur without motor slowing (Additional file 1: Video 1).

Ling and coworkers studied repetitive index finger-to-thumb tapping movements of patients with Parkinson disease and PSP using quantitative motion analysis [35]. They found that the repetitive finger movements in PSP were reduced in amplitude, but there was no decrement. By contrast, repetitive finger movements in Parkinson disease declined in speed and amplitude over a period of 15 s. Ling and coworkers referred to the reduced movement amplitudes as hypokinesia and emphasized that decrements in amplitude and speed are characteristic of Parkinson disease. However, patients with advanced Parkinson disease exhibit repetitive finger tapping that is very reduced in amplitude, and there is little or no decrement [36]. Thus, repetitive finger tapping in advanced Parkinson disease resembles that in PSP [36]. Furthermore, hypokinesia is not defined consistently in the literature and is often defined as paucity of movement (the opposite of hyperkinesia) rather than reduced amplitude [34].

Akinesia literally means absence of movement, and akinesia is commonly viewed as an extreme of bradykinesia [15]. Freezing and festination of gait are commonly regarded as features of akinesia [15] and are associated with festination of speech and repetitive upper limb movements [9]. The motor features of the PAGF phenotype have all been regarded as features of akinesia, and this phenotype illustrates that festination and freezing can occur in the absence of bradykinesia, defined simply as slowness of movement. Freezing also correlates poorly with bradykinesia and rigidity in Parkinson disease [37]. These clinical observations suggest that akinesia should not be considered an extreme of bradykinesia.

## Conclusions

The syndrome of PAGF is most commonly caused by PSP. The PSP-PAGF phenotype illustrates that akinesia is not simply an extreme of bradykinesia because patients with PSP-PAGF initially are not slow. The terms akinesia, bradykinesia and hypokinesia have not been defined and used consistently by clinicians, and it seems advisable to document the occurrence of start hesitation, freezing, festination and decrement without a presumptive label of bradykinesia, hypokinesia or akinesia [34].

## Abbreviations

GPe: Globus pallidus externa
GPi: Globus pallidus interna
MLR: Midbrain locomotor region
MRI: Magnetic resonance imaging
PAGF: Pure akinesia with gait freezing
PNLA: Pallidonigroluysian atrophy
PSP: Progressive supranuclear palsy
SNc: Substantia nigra pars compacta
SNr: Substantia nigra pars reticulata
STN: Subthalamic nucleus

## References

[1] Steele JC, Richardson JC, Olszewski J (1964). Progressive supranuclear palsy: a heterogeneous degeneration involving the brain stem, basal ganglia and cerebellum with vertical gaze and pseudobulbar palsy, nuchal dystonia and dementia. Arch Neurol.

[2] Respondek G, Hoglinger GU (2016). The phenotypic spectrum of progressive supranuclear palsy. Parkinsonism Relat Disord.

[3] Respondek G, Stamelou M, Kurz C, Ferguson LW, Rajput A, Chiu WZ, van Swieten JC, Troakes C, Al Sarraj S, Gelpi E (2014). The phenotypic spectrum of progressive supranuclear palsy: a retrospective multicenter study of 100 definite cases. Mov Disord.

[4] Williams DR, Holton JL, Strand K, Revesz T, Lees AJ (2007). Pure akinesia with gait freezing: a third clinical phenotype of progressive supranuclear palsy. Mov Disord.

[5] Homma Y, Takahashi H, Takeda S, Ikuta F (1987). An autopsy case of progressive supranuclear palsy showing "pure akinesia without rigidity and tremor and with no effect by L-dopa therapy (Imai)". No To Shinkei.

[6] Inzelberg R, Plotnik M, Harpaz NK, Flash T (2016). Micrographia, much beyond the writer's hand. Parkinsonism Relat Disord.

[7] Young WR, Shreve L, Quinn EJ, Craig C, Bronte-Stewart H (2016). Auditory cueing in Parkinson's patients with freezing of gait. What matters most: action-relevance or cue-continuity?. Neuropsychologia.

[8] Nutt JG, Bloem BR, Giladi N, Hallett M, Horak FB, Nieuwboer A (2011). Freezing of gait: moving forward on a mysterious clinical phenomenon. Lancet Neurol.

[9] Vercruysse S, Gilat M, Shine JM, Heremans E, Lewis S, Nieuwboer A (2014). Freezing beyond gait in Parkinson's disease: a review of current neurobehavioral evidence. Neurosci Biobehav Rev.

[10] Elble RJ (2007). Gait and dementia: moving beyond the notion of gait apraxia. J Neural Transm.

[11] Nonnekes J, Snijders AH, Nutt JG, Deuschl G, Giladi N, Bloem BR (2015). Freezing of gait: a practical approach to management. Lancet Neurol.

[12] Factor SA, Jennings DL, Molho ES, Marek KL (2002). The natural history of the syndrome of primary progressive freezing gait. Arch Neurol.

[13] Han S, Oh M, Oh JS, Lee SJ, Oh SJ, Chung SJ, Park HK, Kim JS (2016). Subregional pattern of Striatal dopamine transporter loss on 18F FP-CIT positron emission tomography in patients with pure Akinesia with gait freezing. JAMA Neurol.

[14] Williams DR, Lees AJ (2009). Progressive supranuclear palsy: clinicopathological concepts and diagnostic challenges. Lancet Neurol.

[15] Imai H (1996). Clinicophysiological features of akinesia. Eur Neurol.

[16] Riley DE, Fogt N, Leigh RJ (1994). The syndrome of ‘pure akinesia’ and its relationship to progressive supranuclear palsy. Neurology.

[17] Matsuo H, Takashima H, Kishikawa M, Kinoshita I, Mori M, Tsujihata M, Nagataki S (1991). Pure akinesia: an atypical manifestation of progressive supranuclear palsy. J Neurol Neurosurg Psychiatry.

[18] Compta Y, Valldeoriola F, Tolosa E, Rey MJ, Marti MJ, Valls-Sole J (2007). Long lasting pure freezing of gait preceding progressive supranuclear palsy: a clinicopathological study. Mov Disord.

[19] Quinn NP, Luthert P, Honavar M, Marsden CD (1989). Pure akinesia due to Lewy body Parkinson's disease: a case with pathology. Mov Disord.

[20] Molinuevo JL, Marti MJ, Blesa R, Tolosa E (2003). Pure akinesia: an unusual phenotype of Hallervorden-Spatz syndrome. Mov Disord.

[21] Konishi Y, Shirabe T, Katayama S, Funakawa I, Terao A (2005). Autopsy case of pure akinesia showing pallidonigro-luysian atrophy. Neuropathology.

[22] Factor SA, Higgins DS, Qian J (2006). Primary progressive freezing gait: a syndrome with many causes. Neurology.

[23] Ahmed Z, Josephs KA, Gonzalez J, DelleDonne A, Dickson DW (2008). Clinical and neuropathologic features of progressive supranuclear palsy with severe pallido-nigro-luysial degeneration and axonal dystrophy. Brain.

[24] Pramstaller PP, Salerno A, Bhatia KP, Prugger M, Marsden CD (1999). Primary central nervous system lymphoma presenting with a parkinsonian syndrome of pure akinesia. J Neurol.

[25] Wong JC, Armstrong MJ, Lang AE, Hazrati LN (2013). Clinicopathological review of pallidonigroluysian atrophy. Mov Disord.

[26] Graff-Radford J, Whitwell JL, Dickson DW, Josephs KA (2013). Pallidonigroluysian atrophy associated with p.A152T variant in MAPT. Parkinsonism Relat Disord.

[27] Facheris MF, Maniak S, Scaravilli F, Schule B, Klein C, Pramstaller PP (2008). Pure akinesia as initial presentation of PSP: a clinicopathological study. Parkinsonism Relat Disord.

[28] Dickson DW, Ahmed Z, Algom AA, Tsuboi Y, Josephs KA (2010). Neuropathology of variants of progressive supranuclear palsy. Curr Opin Neurol.

[29] Yoshikawa H, Oda Y, Sakajiri K, Takamori M, Nakanishi I, Makifuchi T, Ide Y, Matsubara S, Mizushima N (1997). Pure akinesia manifested neuroleptic malignant syndrome: a clinical variant of progressive supranuclear palsy. Acta Neuropathol.

[30] Mizusawa H, Mochizuki A, Ohkoshi N, Yoshizawa K, Kanazawa I, Imai H (1993). Progressive supranuclear palsy presenting with pure akinesia. Adv Neurol.

[31] Jahanshahi M, Obeso I, Rothwell JC, Obeso JA (2015). A fronto-striato-subthalamic-pallidal network for goal-directed and habitual inhibition. Nat Rev Neurosci.

[32] Hughes AJ, Daniel SE, Blankson S, Lees AJ (1993). A clinicopathologic study of 100 cases of Parkinson's disease. Arch Neurol.

[33] Postuma RB, Berg D, Stern M, Poewe W, Olanow CW, Oertel W, Obeso J, Marek K, Litvan I, Lang AE (2015). MDS clinical diagnostic criteria for Parkinson's disease. Mov Disord.

[34] Schilder JC, Overmars SS, Marinus J, van Hilten JJ, Koehler PJ (2017). The terminology of akinesia, bradykinesia and hypokinesia: past, present and future. Parkinsonism Relat Disord.

[35] Ling H, Massey LA, Lees AJ, Brown P, Day BL (2012). Hypokinesia without decrement distinguishes progressive supranuclear palsy from Parkinson's disease. Brain.

[36] Bologna M, Leodori G, Stirpe P, Paparella G, Colella D, Belvisi D, Fasano A, Fabbrini G, Berardelli A (2016). Bradykinesia in early and advanced Parkinson's disease. J Neurol Sci.

[37] Bartels AL, Balash Y, Gurevich T, Schaafsma JD, Hausdorff JM, Giladi N (2003). Relationship between freezing of gait (FOG) and other features of Parkinson's: FOG is not correlated with bradykinesia. J Clin Neurosci.

[38] Katayama S, Watanabe C, Khoriyama T, Oka M, Mao JJ, Yamamura Y, Tahara E, Nakamura S (1998). Slowly progressive L-DOPA nonresponsive pure akinesia due to nigropallidal degeneration: a clinicopathological case study. J Neurol Sci.

